# MicroRNAs as Modulators of the Immune Response in T-Cell Acute Lymphoblastic Leukemia

**DOI:** 10.3390/ijms23020829

**Published:** 2022-01-13

**Authors:** Martina Del Gaizo, Ilaria Sergio, Sara Lazzari, Samantha Cialfi, Maria Pelullo, Isabella Screpanti, Maria Pia Felli

**Affiliations:** 1 Department of Molecular Medicine, Sapienza University of Rome, 00161 Roma, Italy; delgaizo.1844853@studenti.uniroma1.it (M.D.G.); sara.lazzari@uniroma1.it (S.L.); Samantha.cialfi@uniroma1.it (S.C.); 2 Department of Experimental Medicine, Sapienza University of Rome, 00161 Roma, Italy; Ilaria.sergio@uniroma1.it; 3 Center for Life Nano Science@Sapienza, Istituto Italiano di Tecnologia, 00161 Rome, Italy; maria.pelullo@uniroma1.it

**Keywords:** microRNA, Notch, Natural Killer cells, T and regulatory T cells, MDSC, Acute lymphoblastic leukaemia

## Abstract

Acute lymphoblastic leukaemia (ALL) is an aggressive haematological tumour driven by the malignant transformation and expansion of B-cell (B-ALL) or T-cell (T-ALL) progenitors. The evolution of T-ALL pathogenesis encompasses different master developmental pathways, including the main role played by Notch in cell fate choices during tissue differentiation. Recently, a growing body of evidence has highlighted epigenetic changes, particularly the altered expression of microRNAs (miRNAs), as a critical molecular mechanism to sustain T-ALL. The immune response is emerging as key factor in the complex multistep process of cancer but the role of miRNAs in anti-leukaemia response remains elusive. In this review we analyse the available literature on miRNAs as tuners of the immune response in T-ALL, focusing on their role in Natural Killer, T, T-regulatory and Myeloid-derived suppressor cells. A better understanding of this molecular crosstalk may provide the basis for the development of potential immunotherapeutic strategies in the leukemia field.

## 1. Introduction

Over the last 30 years, microRNAs (miRNAs) have been the subject of much interest in molecular biology, both for clinical diagnostics and as therapeutic targets in human diseases.

The role that the immune system plays in cancer is now well established, as is the fact that miRNAs act as downstream and upstream modulators that activate important factors such as nuclear factor kappa-B (NF-κB), signal transducers and activators of transcription 3 (STAT3), tumour necrosis factor (TNF), and transforming growth factor β (TGFβ), which tune many immune cell functions [1]. However, the mechanism that regulates the expression of these miRNAs is still unclear.

To date, some 2000 miRNAs are known to be involved in the regulation of 30–40% of the genes in the human genome that regulate all aspects of cell proliferation, differentiation and function [2]. Abnormal gene expression by miRNAs is associated with the progression of cancer, inflammatory diseases or autoimmune diseases. This has shed new light on the mechanisms of interaction between cancer and inflammation [3].

Epidemiological studies suggest that as many as 25% of all cancers may be due to an extrinsic pathway driven by inflammatory conditions and an intrinsic pathway driven by oncogenic alterations that create an inflammatory microenvironment [4].

Immune cells produce various growth factors such as chemokines and cytokines or proteinases that can all create a pro-inflammatory tumour microenvironment. Intercellular communication between cancer and immune cells plays a key role in modulating the immune response, thereby promoting cell migration and proliferation, and tumour progression [5].

The aim of this review is to analyse in depth the miRNAs in the context of Natural Killer (NK), T, T-regulatory (Treg) and myeloid derived suppressor cells (MDSCs) (Table 1), all involved in the crosstalk between the immune microenvironment and cancer cells in haematological malignancies, in particular T-ALL.

**Table 1:** miRNAs involved in ALL immune response.

	T Cells	NK	T Reg	MDSC	T-ALL	Reference
**miR-15-16**	-	Promotes the transition to stage 4 (CD27¯CD11b^+^) during NK maturation	-	-	Blocks NK cell maturation and leads to accumulation of immature stage-2 and stage-3 cells	[6]
**miR-16-2-3p**	-	-	Blocks the function in the tumour microenvironment by inhibiting PD1/PDL1 signalling pathway	Blocks the function in the tumour microenvironment by inhibiting PD1/PDL1 signalling pathway	Down-regulation in ALL leads to the recruitment of both MDSCs and Tregs; inhibits functional T-cell responses in ALL	[7,8]
**Cluster 17-92**	Increases T-cells proliferation and survival in DN–DP transition; high expression leads to autoimmunity	-	-	-	Negatively regulates E2F1 to suppress T-ALL apoptosis	[9,10,11]
**miR-21**	-	-	Induces IL-10 and impairs Treg cell function	-	Up-regulation in T-ALL promotes survival suppressing Pdcd4 at least by stabilization of BCL-XL protein levels; immunosuppressive effects of IL-10 by Tregs leads to unchecked inflammation and promotes T-ALL progression	[12,13,14]
**miR-26**	-	-	Promotes Tregs production and function by targeting IL-6	-	Down-regulation in T-ALL patients leads to decreased apoptosis and induction of proliferation caused by failed repression of PIK3CD	[15,16]
**miR-29a/b**	miR-29a acts as tumour suppressor targeting DNMT3a and DNMT3b to reduce methylation in leukemic T-cells	miR-29b represses NK cells cytotoxic function and differentiation in Notch1-T-ALL	-	-	miR-29b downregulation in T-ALL causes altered epigenetic status activating methylation of DNMT3a, DNMT3b and TET1; miR-29b up-regulation via vesicular release by leukemic cells influences NK cell development; miR-29a low expression in T-ALL	[17,18,19,20]
**miR-34a-5p**	miR-34a acts as tumour suppressor, central NF-κB regulator in T-cell	-	-	Up-regulation of miR- 34a- 5p promotes the expansion of MDSCs	Down-regulation in T-ALL impacts the NF-κB signalosome increasing surface abundance of TCRA and CD3E caused by lost targeting of NF-κBIA and five PKC isozymes	[21,22,23]
**miR-133b**	-	-	Modulates Tregs differentiation by IL-17A expression	-	Reduced expression in association with low levels of IL-17 levels in T-ALL	[16,24]
**miR-142-3p**	Modulated in thymocytes differentiation	-	-	-	Over-expression in T-ALL reduces cAMP levels and interacts with PKA, to increase leukemic T-cell growth; targeting GRa it induces glucorticoid resistance	[25]
**miR-146b-5p**	High expression in thymocytes regulates the DP–SP transition	-	-	-	Tumour suppressor function delaying progression of T-ALL; Downregulated by over-expressed TAL1 in T-ALL patients	[26]
**miR-150**	Modulates T-cell differentiation; regulates Notch3 expression	Promotes NK cell survival and maturation	Up-regulation blocks mTOR pathway and enhances Treg cell differentiation	-	Induces accumulation of hyperfunctional mature NK cells; inversely correlated to Notch3 expression	[27,28,29]
**miR-155**	Positively regulates Th17 differentiation. Pivotal role in regulation of T-cell response during T-cell activation	Promotes the transition to stage 4 (CD27¯CD11b^+^) during NK cell maturation	Targets FOXP3, to modulating Tregs differentiation and function; affects IL-2 production by inhibiting SOCS1 to regulate Treg proliferation	Down-regulation promotes expansion of functional MDSCs by targeting SHIP-1 and PTEN	Blocks NK cell maturation and leads to immature stage 2 and 3 cells accumulation; Low-expression, induced by IL-10, promotes T-ALL progression	[12,30,31,32,33,34,35,36]
**miR-181**	High expression during the DP stage and low mature T-cells	Up-regulates Notch signalling in NK cell development by targeting NLK	-	-	Is a tumour suppressor in ALL with over-expression of Smad7 and regulates TGFβ1/Smad pathway	[37,38,39]
**miR-210**	-	-	Up-regulates FOXP3 and arginase 1, and enhances Treg cells function	Regulates MDSC function by increasing arginase activity and nitric oxide production and, in splenic MDSC, regulates arg1, CXCL12and IL16	Up-regulation of miR-210 in ALL patients may potentiated immunosuppressive activity of tumour MDSCs	[40,41]
**miR-223**	High expression in early thymocytes; down-regulation in DN2–DN3 stage	-	-	Down-regulation represses the differentiation and accumulation of MDSCs by targeting MEF2C	Inhibits the expression of the tumour suppressor FBXW7 and induces T-ALL cell growth in a Notch-dependent manner	[42,43,44,45]
**miR-325**	Controls T-cell proliferation and apoptosis by binding BAG2	-	-	-	Low-expression enhances BAG2 levels in patients with T-ALL and promotes cancer cells proliferation	[46]
**miR-483-3p**	-	Involved in the development and cytolytic function of NK by modulating IGF-1	-	-	Higher expression level plays a positive role in T-ALL pathophysiology	[47,48]
**miR-653-5p**	Enhances apoptosis and autophagy	-	-	-	Negatively regulated by circ-PRKD promotes T-ALL cell proliferation	[49]

This table describes all the miRNAs that have been implicated in the immune response. It describes their expression and function in NK, T and Tregs, and MDSC cells and their deregulation in T-ALL. Keyword: miRNAs, Notch, T cells, Natural Killers (NK), T-regulatory cells (Tregs), Myeloid-derived suppressor cells (MDSCs), Acute Lymphoblastic Leukaemia (ALL), T-cell ALL (T-ALL); PD1: programmed death 1; PDL-1: programmed ligand death 1; DN: double negative; DP: double positive; SP: single positive; E2F1: E2F transcription factor 1; IL-2/IL-6/IL-10/IL-16/IL-17: Interleukin 2/6/10/16/17; DNMT3a/b: DNA (cytosine-5)-methyltransferase 3a/b; cAMP: cyclic adenosine monophosphate; PKA: proteinase Kinase A; PTEN: phosphatase and tensin homolog; SHIP1: Src homology 2 (SH2) domain-containing inositol polyphosphate 5′-phosphatase 1; mTOR: mechanistic target of rapamycin; SOCS1: suppressor of cytokine signalling 1; SMAD7: small mother against decapentaplegic; TGF-β: transforming growth factor beta; FOXP3: forkhead box P3; CXCL12: C-X-C motif chemokine 12; BAG2: BCL-2-associated athanogene; PRKD: protein kinase D; MEF2C: myocyte-specific enhancer factor 2C; IGF1: insulin-like growth factor 1.

## 2. T-Cell Acute Lymphoblastic Leukaemia 

Acute Lymphoblastic Leukaemia (ALL) is a heterogeneous disease that causes malignant haematological disorders at any age. It mainly affects children aged 2 to 5; in fact, 60% of paediatric leukaemia cases are ALL, with an incidence of 3–4 cases per 100,000 per year. It is divided into two subtypes B-ALL and T-ALL depending on whether transformation occurs in B- or T-cell precursors, respectively [50]. 

The evolutionarily conserved Notch signalling pathway plays a critical role in cell fate choices in many tissues and its dysregulation contributes to the development of various malignancies, including T-ALL. Four Notch paralogues (Notch1–4) in mammals bind transmembrane ligands of the Jagged family (Jagged-1, Jagged-2) or the Delta-like family (DLL1, DLL3 and DLL4) [51]. Through a multistep proteolytic process, the intracellular domain of Notch (NICD) is released and translocated into the nucleus where it complexes with the DNA-binding CSL/RBP-Jk factor, the Mastermind-like co-activator (MAML1–3) and other nuclear factors to form the Notch transcriptional complex that then regulates the transcription of multiple genes [52,53].

Loss-of-function or gain-of-function experiments suggest that Notch blocks B-cell differentiation by inducing lymphoid progenitor cells towards T-lineage differentiation. In the absence of the Notch signalling pathway, progenitor cells enter the thymus and differentiate into the B-cell lineage. In contrast, when the receptor is constitutively expressed there is inhibition of B-lineage differentiation with an accumulation of T-cells [54]. Indeed, constitutive activation of Notch signalling pathway drives to T-ALL, whereas potent activation of Notch signalling exerts pro-apoptotic effects in B-ALL cells [50].

Thymocyte differentiation begins when bone marrow-derived lymphoid progenitors enter the thymus at the corticomedullary junction and the chemokine receptor CXCR4 (chemokine receptor C-X-C type 4) progressively drives their differentiation into mature single-positive thymocytes (SP; CD4^+^ CD8^+^), fully competent for immune response in the periphery [55]. Both Notch1 and Notch3, in association with several nuclear factors, regulate these early stages of thymocyte progenitors differentiation [54,56,57]. 

T-cell acute lymphoblastic leukaemia (T-ALL) is an immature lymphoid tumour characterised by infiltration of the bone marrow by malignant haematopoietic cells expressing immature T-cell markers. The incidence of T-ALL cases is 15% in children and 25% in adults and is slightly more frequent in males than in females. Relapses of paediatric T-ALL remains a significant clinical problem [58]. 

T-ALL is the result of a multistep transformation in which accumulating genetic alterations co-ordinately disrupt master developmental pathways responsible for the normal control of immature T-cell growth, proliferation, survival and differentiation [59].

Deregulation of the NOTCH1 or NOTCH3 signalling pathways is intimately involved in the pathogenesis of T-ALL [60,61] and greatly supported by partnership with CXCR4 [62].

More than 50% of patients with T-ALL have NOTCH1 mutations and MYC has been identified as a key oncogenic mediator of NOTCH1 [63]. Overexpression and more recently activating mutations of Notch3 have been found in a high number of human T-ALL samples [64]. Therefore, targeting Notch is one of the major challenges [65], and treatment strategies that minimize the need for chemotherapy or other targeted anti-leukaemia agents are highly desirable.

## 3. Overview of the Immune Mechanisms in T-ALL

In T-ALL development, abnormal T-lymphoid progenitors invade bone marrow, peripheral blood, and extramedullary sites [50]. The interaction of leukemic cells with the microenvironment within the bone marrow niche is a critical step promoting the progression of T-ALL. The triggering of multiple molecular mechanisms shapes immune cell populations involved in the immune surveillance, such as NK cells, various population of T-cells, including regulatory T-cells (Tregs) and MDSCs [66,67]. 

NK cells are a population of immune cells that coordinates both the innate and the adaptive response; they constitute 5–15% of circulating lymphocytes and in various lymphoid and non-lymphoid organs [68]. NK cells have the ability to identify, target and kill cancer cells without prior sensitization. BM is considered to be the primary site of NK development but over the years many studies have reported that NK cells can also develop in secondary lymphoid tissue [68]. The balance between the activating and inhibitory receptors on NK cells and the cognate ligands on ALL cells determines the ability of NK to kill ALL blasts. To escape from NK cell lysis ALL blasts predominantly down-regulate ligands for NK cell-activating receptors [67,69]. Patients suffering from ALL have abnormal percentages and absolute numbers of NK cells, as confirmed especially in those diagnosed with T-ALL [70]. More recently, an interesting paper reported that in high-risk patients with B/T-ALL NK cells are unable to lyse NK-sensitive targets with the same efficiency of normal NK cells because have a defective maturation into cytotoxic effectors.. Moreover, an increased frequency of activated cytokine-producing NK cells is associated with disease severity and poor clinical outcomes [71]. Therefore, immunotherapy using NK cells derived from healthy donors may be a more effective therapeutic option, and ongoing protocols try to optimize in vitro NK cell expansion [72].

T-cells are not as effective against cancer as expected, partially because they enter a dysfunctional or exhausted state, mostly triggered by tumour cells, Tregs and MDSC [73]. This may imply sustained expression of inhibitory receptors and a transcriptional program different from that of functional effector and memory T-cells [73]. Indeed, in T-ALL attenuation of T-cell-mediated antitumour immune response may rely on the expression of inhibitory checkpoint receptors on conventional T cells and on the accumulation and increased suppressive function of Tregs [74]. 

The involvement of PD1 (programmed death 1) and PDL1 (programmed death ligand 1) in T-ALL is still rarely reported [75]. PD1 expression was observed in tumour infiltrating lymphocytes in approximately 20% of T-LBL/ALL patients, but absent on their tumour cells of T-LBL/ALL. In contrast, these tumour cells and activated lymphocytes in the reactive lymph nodes from the T-LBL/ALL tissues showed a positive result for PDL1 [75]. Therefore, PDL1 could negatively regulate T-cell activation by binding PD1 to the surface of tumour infiltrating immune cells and promote immune escape.

Furthermore, there are other inhibitory interactions involving leukemic T-cells, such as TIM-3 and its ligand, galectin-9, acting in the inhibition of CD8^+^ T cell responses in early T-cell precursor ALL [76]. Additionally, in T-ALL even more challenges for strong T-cell-mediated immune response/immunotherapies are met [77]. As suggested by Pastorczak et al., this can be partly due to the overlap of target antigens between leukemic and normal T-cells, possibly leading to off-target effects [67]. 

CD4^+^ CD25^+^ FoxP3^+^ Tregs, previously known as suppressor T-cells, are immunoregulatory subset of T-lymphocytes that play a crucial role in the maintenance of tolerance to self antigens and the modulation of overall immune responses against infections and tumour cells [46,78,79]. A dysregulation or an excess of Tregs could lead to a large type of immune-related diseases and cancers. The regulatory role of Tregs is mediated by various mechanisms: the secretion of the inhibitory cytokines, such as interleukin-10 (IL-10), interleukin-35 (IL-35) and TGFβ; cytolysis of target cells directed by granzyme-A, granzyme-B, and perforin [80]. The potential role of the Notch/NF-κB partnership is also emerging in the generation and function of Tregs in the context of cancer, and more precisely in T-ALL [81]. In haematological malignancies, Treg accumulation is associated with increased tumour progression and suppression of anti-tumour immune responses [82,83]. Rare studies have provided a correlation of Treg number with disease progression and explained how they elicit such a potent suppressive mechanism. Additional functional studies are indeed required. Hence, strategies focused on Treg ablation or selective inactivation are key elements in combination therapies against T-ALL.

In the leukemic microenvironment, macrophages play a role as immune effector cells. Myeloid cells are essential for the homeostasis of the innate and adaptive immune response [84,85]. The pro-tumorigenic function of MDSC is well established in solid tumours and some haematological malignancies, but their role in T-ALL is still poorly understood.

Cancer immunotherapy, which triggers or augments host immune responses to treat haematological malignancies, is the rapidly advancing and innovative approach to cancer immunology. 

Tumour-infiltrating NK cells, T and Treg cells, myeloid cells, including MDSCs are major obstacles to the development of successful cancer immunotherapies. In this regard, in-depth miRNA studies could improve specific immune cell expansions and selective targeting of key components of the immune system to increase anticancer response and to avoid immune surveillance evasion strategies by T-ALL cells.

## 4. Biogenesis and Role of miRNAs

MiRNAs are a class of single-stranded (ss) non-coding RNAs 22 nucleotides long. By binding to the 3’ untranslated regions (UTRs) of the target mRNA, they regulate the post-transcriptional gene expression of target genes by influencing the cellular processes to which they are involved. 

MicroRNA genes are usually non-coding and found throughout the genome. In some cases, they are also present in introns or in an UTR of a protein coding gene, but generally they are not found in coding exons as its excision would lead to the loss of the protein coding transcript [86].

Starting from a DNA template, RNA polymerase II begins the transcription of an RNA strand, analogous to mRNA, defined as primary miRNA (pri-miRNA). 

Primary miRNAs are similar to mRNAs with the cap and polyadenylate sites, but they are also characterized by a hairpin structure recognized by a complex formed by RNase III Drosha and the Di George syndrome critical region gene 8 protein dimer (DGCR8), which recognizes the hairpin stem and cleaves the pri-miRNA producing a pre-miRNA [87].

The structural change allows the binding of the pre-miRNA to the nuclear transport receptor Exportin 5 and Ran GTPase, which translocate the pre-miRNA from the nucleus into the cytoplasm [88]. During the cytoplasmic phase, the DICER enzyme cleaves the precursor loop and forms RNA duplex of ~21 nucleotides, depending on the miRNA. One strand is loaded into the RISC complex to become mature miRNA, while the other strand is present in the cytoplasm at a lower concentration compared to the guide strand; despite the low concentration, it may also be incorporated into the RISC complex or degraded [89]. 

The peculiarity of miRNA is the seed sequence: a segment from 2 to 8 nucleotides at 5′ complementary to the response element of the miRNA (MRE) located in the 3’UTR of the mRNA. Different mRNAs can have the same MRE and therefore be targets of the same miRNA.

If complementarity is high, miRNA induces degradation of the target mRNA, while if homology is less extensive, they induce translation repression [13].

Over the past decade, many studies have focused attention on the role of miRNAs in cancer––particularly tumour growth, angiogenesis, invasion and immune evasion––by controlling the expression of target mRNAs [90]. Depending on their expression in tumor cells and their role, miRNAs can be divided into two categories: oncogenic-miRNAs and suppressor miRNAs.

Oncogenic miRNAs (onco-miR) are up-regulated in tumour cells and contribute to carcinogenesis by inhibiting tumour suppressor genes. Suppressor miRNAs are down-regulated in tumour cells and normally prevent cancer development by inhibiting the expression of proto-oncogenes. 

We will now discuss the role of miRNAs in the development of immune cells (NK, T-cells, Tregs and MDSCs) and their deregulation (Table 1) as a potential mechanism of impaired immune response that possibly sustains the maintenance and progression of T-ALL. 

## 5. miRNA and Natural Killer (NK) Cells

### 5.1. miRNA in NK Cell Development: An Overview

The development of NK cell progenitors progresses through different stages: Stage 1 cells express CD34 but lack CD117, CD94, NKP80, CD16 and the IL-2/IL-15 receptor beta chain [28]. Stage 2 cells are characterized by CD25 (IL-2 receptor), CD122 and CD117. During these two stages, cells also have the capacity to develop into T-cells and dendritic cells (DCs). Stage 3 cells lack the expression of TBX21 (TBET) and Eomesoderm (EOMES) [68]. The transition of NK cells from Stage 2 to Stage 3 is promoted by the expression of activation receptors including NKG2D (Natural Killer Group 2D), CD335, and CD337 [91]. Stage 4 NK cell development is divided into 4a and 4b and identified by the expression of the activating receptor NKp80 [92]. In Stage 4a, the NK cells are NKp80^−^ CD56^bright^, while at Stage 4b, the NK cells become positive for NKp80 and maintain their CD56^bright^ status. In fact, the terminal stages of NK cell development in the blood are dependent on the relative expression of CD56, with the distinction between immature CD56^bright^ and functionally mature CD56^dim^ NK cell subsets [91]. 

To explicate their complete function, NK cells undergo an educational process by contacting “self” major histocompatibility complex (MHC) class I receptors [93]. During tumour invasion, cancer cells down-regulate MHC class I to evade T-cell recognition; this leads to NK cell engagement and activation because of the lack of inhibitory stimulation from binding to killer-cell immunoglobulin-like receptors (KIRs), also called the “missing self” hypothesis [94]. 

NK cell development is affected by various miRNAs such as miRNA-15/16, miRNA-155, miRNA-181 and miRNA-483-3p [28]. In leukemic cell survival, miRNAs-15/16 is inversely correlated with the levels of oncogenes, such as B-cell lymphoma 2 (Bcl2), which is expressed in several haematological diseases [6], myeloid cell leukemia-1 (MCL1), JUN, and WNT3a [95].

Sullivan et al. demonstrated that mice with global deletion of miR-15a/16-1 had defective NK cell maturation with a block in the most mature Stage 4 of murine NK cells (CD27-CD11b +) and an associated build-up of immature cells (Stage 2 and 3) [96].

This increased amount of immature NK cells was also demonstrated by Trotta et al. using a murine transgenic model with miRNA-155 overexpressed under a lck promoter. These authors showed that higher miRNA-155 expression led to an increase in splenic NK cell numbers with an excess of immature (CD11b^−^CD27^+^) NK cells with high cytokine generation but low cytolytic activity [34]. As previously described, NK cell survival and maturation are under the control of miRNA-150 and miRNA-181; in fact, mice that overexpressed miRNA-150 had an accumulation of mature hyperfunctional NK cells that may lead to NK cell exhaustion [97].

miR-181 has a central role in regulating the differentiation of B-cells, T-cells and NK cells during normal haematopoiesis [37]. NK cell maturation is promoted by Notch signalling via CD56 acquisition and can also bypass the need for stroma or cytokines like IL-15 to drive NK cell maturation [98].

It has been demonstrated that miR-181 is able to modulate NK cell development through the regulation of Notch signalling by targeting Nemo-like kinase (NLK), an inhibitor of Notch [98]. NLK is regulated by miR-181 specifically on the 3′-UTR. Overexpression of miR-181 down-regulates NLK expression but conversely promotes NK cell development through the Notch signalling pathway. Indeed, miR-181 is also a positive regulator of IFN-γ, which together with the negative regulator miR-146, balances its expression in NK cells during the immune response [37].

NK cell development could also be modulated by miRNA-483-3p through the downregulation of insulin-like growth factor-1 (IGF-1). Indeed, high exogenous IGF-1 is linked with increased perforin expression in the NK cell, which is critical for cytolytic NK cell function [99]. 

### 5.2. miRNAs Modulate NK Cell Function in T-ALL

For most of the time, the study of NK cells in the field of leukaemia has been limited to acute myeloid leukaemia (AML) and B-ALL [19]. Little information is related to T-ALL; in fact, some immunotherapies based on NK cells have been proven effective in clinical treatments, especially in AML, but are less promising for T-ALL. The reason is not totally understood, but could be because T-ALL blasts are better for contrasting NK cell cytotoxicity [19]. 

Although there are more than 100 miRNAs involved in leukemic cells alterations, only few of them directly trigger functional and developmental dysregulation of NK cells. NK cells are important innate immune surveyors that are critical for the removal of leukaemia blasts [100]. T-ALL blasts lead to a reduction on the cell surface of ligands that normally bind NK cell receptors, thus allowing the cells to escape more easily from immunosurveillance control [101].

The biological functions of NK cells and their development are often subject to regulation by microRNAs, and small non-coding RNAs (sncRNAs). The former dysregulate NK cell development and maturation through the deletion of key enzymes in the miRNA biogenesis pathway, such as Dicer and DGCR8. In so doing, mature miRNAs are critical for normal NK-cell homeostasis and activation [102].

For example, microRNA-29b (miR-29b) up-regulation was identified in NK cells in both neurogenic locus notch homolog protein 1 (Notch1)–T-ALL mice and patients with T-ALL. This miRNA is involved in diseases with a severe phenotype; furthermore, it affects the development of NK cells [103].

The expression of miR-29b is higher in NK cells than the level found in T-ALL blasts. This increase is probably due to the vesicular/exosomal release of miR-29b by leukemic blasts as the disease progresses [103]. Further studies of miRNA-29b regulation are needed to fully understand this phenomenon.

Increased levels of miRNA-29b promote decreased cytotoxicity by NK cells in T-ALL. In addition to functionality, NK cell differentiation is also impaired in Notch1–T-ALL and contributes to reduced cytotoxicity by NK cells [18]. 

Therefore, miRNA-29b turns out to be a crucial partner for leukemic cells in their escape from the surveillance system in T-ALL.

The oncogene Wilms’ Tumor 1 (WT1), normally overexpressed in ALL, is also a potential indirect target of miR-15a and miR-16-1; lowering WT1 gene levels promotes the proliferative blockade of leukemic cells [17]. 

On the other hand, miRNAs-15/16 play a key role in the maturation of NK cells and the expression of their function; in this way, they support both the reduction of NK cell cytotoxic capacity and the anti-leukemic effect [95].

Two Notch3 targets, miR-150 and miR-223, regulate NK cell survival, maturation [104] and immune function [68] or specifically control Granzyme B translation in resting NK cells [97], respectively. This would suggest a role for Notch in modulating the immune response in T-ALL.

miR-181a could act as a tumour suppressor in paediatric ALL by over-expressing its target, small mother against decapentaplegic 7 (Smad7) [38], which regulates TGF-β1 via negative feedback and mediates the interaction between TGF-β1 and other pathways. Moreover, TGF-β1 had a role in NK dysfunction in B-ALL blasts, which secrete TGF-β1 and use the TGF-β1/Smad pathway to inhibit NK cytotoxicity [105].

In this way, the functional miR-181–Smad7–TGF-β1 relationship may contribute to the discovery of new targets for ALL diagnosis and therapies [38].

Alterations in NK cell maturation and function resulted in decreased NK cytotoxicity in T-ALL, suggesting that these miRNAs are involved in NK cell developmental arrest and functional defects, which are used by leukaemia cells to evade immune surveillance. Further research is required to reveal the mechanism driving this phenomenon and for solutions that restore NK cell maturation and function to decrease T-ALL relapse.

The regulation of NK cell maturation and function by miRNAs may be one of the suitable therapeutic mechanisms for promoting anti-leukemic efficacy.

## 6. miRNAs and T Cell 

### 6.1. miRNAs in T Cell Development: An Overview

Many excellent articles have revised the role of miRNAs as key regulators in gene expression in immunity and in T-cell development [106,107]. T-lymphocytes, or T-cells, arise from the early T-lineage precursor of the bone marrow that is guided in the thymus to mature and then migrate into peripheral immune tissues to carry out biological functions [106]. In the thymus, the central immune organ, the development of T-cells is divided into several stages according to the expression of CD4 and CD8 coreceptors [108]. 

Starting from the cortical region, early T-cells gradually differentiate into CD4^–^CD8^–^ double-negative (DN) cells, and then subsequently divided into four stages (DN1-2-3-4). Under the control of Notch, DN T-cells develop into CD4^+^ CD8^+^ double-positive cells (DP), with the expression of the T-cell receptor (TCR), and gradually migrate into the medulla. Mature T-cells express only CD4 or CD8 coreceptors to become single-positive (SP) CD4^+^ or CD8^+^. At this point, they migrate to the peripheral lymphoid organs to perform their immune functions. T-cell immunity is influenced by microRNAs that act on the development and function of immune system cells and on related clinical diseases [106].

The miR-17-92 cluster is highly modulated at DN Stage 1 leading to autoimmunity and increased proliferation and survival of T-cells, above all the effector CD4^+^ T-cells [11]. Activation of miR-17-92 by NK like homeodomain proteins suppresses apoptosis in T-ALL by reducing the level of E2F transcription factor 1 (E2F1) [9]. The miR-17-92 cluster blocks the expression of phosphatase and tensin homolog (PTEN) and the pro-apoptotic Bcl-2-like protein 11 (BIM), thereby promoting T-cell survival at the DN2 stage. Furthermore, T-cell survival from the DN to DP stage is influenced by miR-17-92, which regulates IL7R receptor surface expression and response to IL-7 [10].

miR-150 is a hematopoietic-specific miRNA. T-cell development is affected by miR-150, which is overexpressed in mature T- and B-cells. In fact, its expression is at maximum level during the last stages of both B- and T-cell maturation in the bone marrow and thymus, respectively, suggesting that it may participate in B-or T-lymphopoiesis [109].

This miRNA was widely investigated in T-ALL; first, for its role during T-cell development through different stages from DN CD4^−^CD8^−^ to T-helper [109]; moreover, because miR-150 targets Notch3, its activating mutations are the leading cause of T-ALL [27,64,110].

The unusual expression of miR-150 has been observed in different haematological malignancies: decreased in lymphoma, chronic myeloid leukaemia (CML) and ALL but increased in chronic lymphocytic leukaemia (CLL) and myelodysplastic syndrome [111]. Indeed, miR-150 exhibited very low expression levels in several T-ALL cell lines. In a study by Ghisi et al., forced expression of miR-150 demonstrated downregulation of Notch3 transcript and dysregulation of biological phenomena, such as inhibition of cell proliferation and induction of cell apoptosis, suggesting its prominent role in T-ALL pathogenesis. 

The miR-146b-5p is another of the miRNAs found at high levels in thymocytes in the transition between the DP (CD4^+^ CD8^+^) to SP stage (CD4^+^ or CD8^+^) [26]. 

miR142-3p is overexpressed in human acute leukaemia T-cells (Jurkat, MOLT-3, MOLT-4) compared to normal counterparts; it has been identified as a haematopoietic-specific miRNA, as it is expressed at different levels of the differentiation stage [25]. The levels of miR-142-3p in leukemic T cells correlate with the prognosis of patients with acute leukaemia. Therefore, its use as a marker in disease outcomes may be advantageous.

The miRNA142-3p also promotes the reduction of cyclic adenosine monophosphate (cAMP) levels and, in this way, interacts with the protein kinase A (PKA) pathway, inducing increased growth of leukemic T-cells. High expression of miR-142-3p characterizes conventional T-cells but not forkhead box P3 (Foxp3^+^) Treg cells; in effector T-cells the miRNA is down-regulated, compared to naive or memory T-cells [25].

miR-223 is among the most up-regulated miRNA in T-ALL [44], and it is a direct target of TAL1 and the Notch–NF-κB axis [43]. TAL1/SCL is part of basic helix–loop–helix (bHLH) family and its expression is aberrant in 60% of human T-ALL cases [45]. Inhibition of miR223 prevents T-ALL resistance to γ-secretase inhibitor treatment, suggesting that this inhibition may be exploited in target therapy protocols.

It has been demonstrated that the expression of TAL1 and miR-223 are modulated during the normal T-cell development; miR-223 expression is high in early thymocytes with a down-regulation after the double-negative-2 (DN2) stage of maturation. Furthermore, miR-223 mediates overexpression of TAL1-induced growth of T-ALL cells through the direct inhibition of the expression of the tumour suppressor F-box/WD repeat-containing protein 7 (FBXW7), which has been shown to repress MYC, MYB, NOTCH1, and CYCLIN E expression [112].

One of miR-181 genes family (mir-181ab1, mir-181ab2 and mir-181cd), miR-181ab1 could control how NOTCH acts in tumorigenesis; indeed, miR-181ab1 deletion has an inhibition effect on T-ALL cells. The expression of miR-181a is high at the DP T-cell stage but decreases during development, with almost no expression in differentiated T-cells [113]. Moreover, the effects of this deletion are compensated for during normal thymic progenitor development but not during T-ALL development [45].

### 6.2. miRNAs Dysregulate T Cell Immune Response in T-ALL

As we know, microRNAs are involved in both functional and maturational regulation of several immune cells, in particular T-cells. Several miRNAs are up- and down-regulated during the differentiation process of thymocytes and during related diseases, including ALL [114]. The definitive understanding of their role in T-ALL is still under study.

Among the miRNAs described, it is now clear that miR-29a plays a key role in haematological diseases, such as AML, CLL and T-ALL, albeit less thoroughly [20]. Indeed, this miRNA appears to play a role in leukemogenesis [99], specifically in the pathophysiology of T-ALL.

In 2015, a study by Oliveira et al., allowed a more detailed approach of miRNA-29a with leukemic T cells, which present a very low expression of miR-29a [20]. 

This miRNA turns out to be very functionally complex: it has DNA methyltransferases DNMT3a and DNMT3b as targets, leading to reduced methylation, but also Cell division protein kinase 6 (CDK6), Peroxidasin-like protein (PXDN), MCL1, CXXC6 (C–X–C motif chemokine receptor 6) in various tumour types and in T-ALL; on the other hand, it is involved in active demethylation through ten-eleven translocation (TET) and Thymine-DNA glycosylase (TDG) proteins [81,114]. The family of TET genes (TET1, TET2, and TET3) have been implicated as tumour suppressors in haematological malignancies [115]. 

Several genes commonly methylated in T-ALL are indeed targeted for demethylation by the action of miRNA [114]. These findings could serve as an input to find new miR-based therapies for leukemias.

One of the first miRNAs discovered in T-ALL pathology is the miR-17-92 cluster, which includes miR-17, miR-18a, miR-19a, miR-20a, miR-19b-1, and miR-92a. Their primary role in leukaemia is to target some of the oncosuppressor genes most involved in T-ALL, such as PTEN, cell cycle inhibitor p21 (CDKN1A) and BCL211 (or BIM) [49].

This miRNAs cluster is overexpressed in T-ALL samples compared with its T-cell counterpart; other miRNAs, which target key T-ALL genes, are differentially modulated, such as miR-223, miR-142-3p, miR-150, miR-93, miR-26a, miR-16, and miR-342 [116].

In T-cell acute lymphoblastic leukaemia for example, the cooperation between miR-19 and Notch1 activation has been observed [116]. Indeed, one study reported that the NOTCH1 gene translocation t(9;14)(q34;q11) was coexistent with the miR-17-92 cluster t(13;14)(q32;q11) in a leukemic clone. The crosstalk between Notch1 and miR-19 is supported by the correlation between the high number of activating mutations in the Notch1 gene in more than 50% of leukaemia patients, and the high level of miR-19 in the same patients in T-ALL [117]. 

miR-19 is a key oncogene in the 17-92 cluster, contributing to the pathogenesis of T-cell leukaemia. In addition, it participates in leukemogenesis, due to its implication in the survival program of T-lymphocytes, via the Phosphoinositide 3-kinases (PI3K) pathway [49]. PI3K/AKT/mTOR (mechanistic target of rapamycin) pathway is inactivated by the depletion of Circ-PRKDC, a circular (Circ) RNA that has been suggested as a participant in the regulation of cell progression in hematopoietic compartments and is known to interact with RNAs and proteins in malignant conditions. Circ-PRKDC also prevents proliferation and induced autophagy and apoptosis. Circ-PRKDC interacts negatively with miR-653-5p, and the restoration of miR-653-5p prevents T-ALL cell proliferation, while enhancing apoptosis and autophagy [118].

miR-146b-5p is regulated by TAL1, a transcription factor essential for the maintenance and regulation of haematopoiesis. TAL1 is overexpressed in more than 60% of patients with T-ALL [111]. In so doing, TAL1 inhibits miRNA 146b-5p, constituting a suppressor for solid tumours and hematologic cancers, including T-ALL. The correlation between miR-146b-5p and TAL1, contributes to the pathogenesis of T-ALL, influencing cell migration and leukaemia aggressiveness, but regarding the proliferation of leukemic T cells, there are no significant effects [119]. T-cell proliferation and apoptosis are also under the control of the expression of miR-325. It is markedly lower and the level of Bcl-2-associated athanogene (BAG2) is markedly higher in patients with T-ALL and in T-ALL cell lines; BAG2 knockdown can influence cell proliferation and apoptosis and miR-325 shares a binding site with BAG2. miR-325 has an inhibitory effect on the proliferation of cancer cells. Additionally, the biological effects of miR-325 on the proliferation and apoptosis of Jurkat cells were reversed by the introduction of BAG2 [46].

In the context of T-cell leukaemia, an attractive hypothesis is that leukemic cells could release exosome-directed miRNAs intending to suppress specific immune cells. This could be suggested by the expression of some miRNAs, such as the Notch3-regulated miR-150, common to T, Treg, and NK cell microenvironments, or the case of miR-29 and miR-181 common to T and NK cells. Indeed, this mechanism has been suggested since miR150 is abundant in plasma-derived exosomes in the microenvironment of AML and CLL patients [78,79] and is possibly associated with impaired NK cell function [97]. 

### 6.3. The Modulatory Role of miRNAs in Treg Cell Function in T-ALL

The transcription factor Foxp3 is highly and specifically expressed in Tregs, which are characterized as the CD4^+^ CD25^+^ Foxp3^+^ T lymphocytes [120]. 

In 2006, Cobb et al. identified 35 microRNAs that were up-regulated in Tregs, including miR-223, miR-146, miR-21, miR-22, miR-23a and b, miR-24, miR-214, and miR-155 and 33 microRNAs that were down-regulated, including miR-142-5p and miR-142-3p, miR-30b, miR-30c, miR-30e [121].

miR-155 targets FOXP3, which might regulate Treg cell differentiation and function. One study refers the reduction in miR-155 level in ALL patients compared to controls; particularly, miR-155 can act as a modulator of Treg cells differentiation by targeting Suppressor Of Cytokine Signaling 1 (SOCS1) [36]. In so doing, miR-155 could affect IL-2 signalling by inhibiting SOCS1 expression to regulate Treg proliferation [31]. Loss of miR-155 can also contribute to B-cell malignancies [33,35].

Several cytokines contribute to Treg regulation and differentiation, including IL-6, IL-17, IL-23, Tumor necrosis factor α (TNF-α), TGF-β and IL-10 [12]. Through the inhibition of programmed cell death protein 4 (PDCD4), IL-10 can specifically down-regulate miR-155; on the other hand, IL-10 can be induced by miR-21 [12]. Knowing that IL-10 and miR-21 are significantly elevated in ALL patients, it was suggested that the slight elevation in Treg cells in the patients’ group might be tightened as a result of the reduction of miR-155. Treg cells exert their immunosuppressive effects via IL-10 release leading to inefficient control of inflammation, which favours paediatric ALL progression [12].

miR-26a and miR-133b are down-regulated in ALL patients compared to controls. miR-26a is found to promote Treg production and up-regulate Treg cell function by targeting IL-6. This is an inflammatory cytokine that has a crucial role in the inhibition of Treg functions [16], while miR-133b was reported within a cluster of miR-206 to be responsible for IL-17A gene co-expression [24]. These data presented by Haas et al. (2011) agree with the reduction in both miR-133b and IL-17 expression levels in ALL patients [122]. 

The cytokine alteration induced by miRNAs during the differentiation of Treg cells could possibly be involved in the pathogenesis of ALL. Other investigations are needed to get a better understanding of the pathologic role of selected miRNAs and their interplaying role with pro-/anti-inflammatory cytokines in ALL.

## 7. MDSCs and Tregs 

MDSCs and Treg cells are immunoregulatory cells of the tumour microenvironment in solid tumours and haematological malignancies [123,124].

Only a few studies have characterized MDSC and Treg cells based on some miRNA expression profiles in cancer, and were recently reviewed in [125]. This could be useful for identifying reliable biomarkers in cancer, particularly in ALL [124].

**Figure 1:**
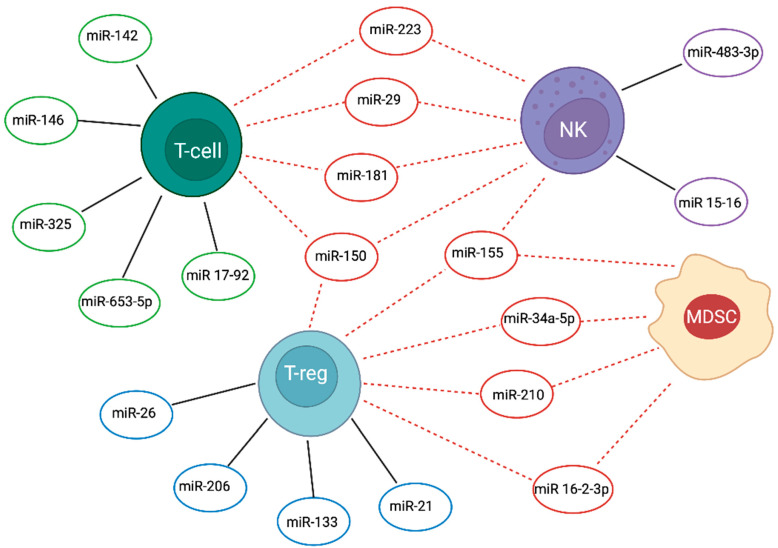
Common and cell-specific miRNAs in immune cell responders against ALL. Common (dotted-line—) and cell-specific (black line—) miRNAs are depicted in the figure in association with the Natural Killer (NK), T and T-regulatory (Treg), and myeloid derived suppressor (MDSC) cells. Created by Biorender.com.

A study found that 12 miRNAs (17.4%) are common among MDSC and Treg cells in ALL patients (miR-10a-5p, -16-2-3p, -21-3p, -21-5p, -145-5p, -146a-5p, -150-3p, 155-5p, -155-5p, -210-3p, -210-5p). They might be involved in 4 immune-related pathways: TNF, TGF-β, Forkhead box O (FoxO) and Hippo. Moreover, some of these common miRNAs modulate the differentiation and the immunomodulation functions of MDSC and Treg cells [7], or even NK maturation and cytolytic activity as is the case of miR-155 [34] as shown in Figure 1. For example, miR-16-2-3p seems to block the function of MDSC and Treg cells in the tumour microenvironment via the inhibition of the PD-L1/PD-1 signalling pathway [8]. 

Up-regulation of miR-210 in pediatric ALL patients may be linked with the miR-210-modulated immunosuppressive activity of tumor MDSCs by increased arginase activity and nitric oxide production, as induced by hypoxia [41,126].

In addition, up-regulation of miR-34a-5p promotes the expansion of MDSCs and the mechanism through which cell surface-associated MUC1 (Mucin 1, cell surface associated) drives c-Myc expression during the leukemogenesis in AML [23]. This is in contrast with a study by Salem et al., where miR-34a-5p was found to be down-regulated in ALL [7]. 

Targeting MDSC can represent a future challenge in modulating immunosuppressive functions in tumour microenvironment.

Still poorly understood is the role of MDSC in ALL, but the acquisition of more knowledge about miRNA tuning could improve the possible application as a therapy.

## 8. Conclusions and Future Perspectives

Some miRNAs have been shown to regulate both lymphoid and myeloid cell lineage of the haematopoietic system and the key role of miRNA-mediated gene regulation has been established in the immune system. However, there are still many unresolved questions concerning how immune cells modulate this process during the anti-leukaemia immune response and how their deregulation may contribute to leukemogenesis. Additionally, the effect of one miRNA on many transcripts of genes often with related function gives complexity to the system. 

The expression signatures of miRNAs are distinct in the differentiation and maturation of the haematopoietic cell lineage and this may be useful in distinguishing Acute Lymphoblastic Leukaemia from Acute Myeloid Leukaemia with 97% accuracy [127]. Moreover, evidence suggests that miRNAs can be used as potential diagnostic and prognostic biomarkers in B-ALL [128]. Diagnostics for haematological malignancies are performed on peripheral blood and bone marrow samples and are based on cytogenetic analysis of the morphological, cytochemical and immunophenotypic characteristics of the cell lineage. To date, in comparison to B-ALL, no miRNA has prognostic or diagnostic value in T-ALL, which is an intensive area of research. Notwithstanding its side effects, intensive chemotherapy still represents the best therapeutic option for T-ALL. Therefore, the characterization of the leukemic microenvironment concerning miRNA-based modulation of immune responses could pave the way for a combination of miRNA-related immunotherapies with conventional cytotoxic drugs or targeted therapy. This is also a valuable opportunity for effective therapeutic intervention for relapsing patients of T-ALL. 

To be further analysed, is the release mechanism of selected miRNAs in the extracellular space, thus contributing to the ability of T-ALL cells to control trafficking of regulatory molecules within the leukemic microenvironment. This intercellular crosstalk could be the way to modulate immune cell function and be a new approach to target anomalous immune response in leukaemia. Accordingly, exosome- and immune cell-based delivery could represent two interesting strategies for miRNA-based cancer immunotherapy.
